# The complex cascade of cellular events governing inflammasome activation and IL-1β processing in response to inhaled particles

**DOI:** 10.1186/s12989-016-0150-8

**Published:** 2016-08-12

**Authors:** Virginie Rabolli, Dominique Lison, François Huaux

**Affiliations:** Louvain Centre for Toxicology and Applied Pharmacology (LTAP), Institut de Recherche Expérimentale et Clinique (IREC), Université catholique de Louvain, Avenue Mounier 52, B1.52-12, 1200 Brussels, Belgium

**Keywords:** Inflammasome, Alarmins, Cytokines, Macrophages, Silica, Nanoparticles, IL-1 family, Inflammation

## Abstract

The innate immune system is the first line of defense against inhaled particles. Macrophages serve important roles in particle clearance and inflammatory reactions. Following recognition and internalization by phagocytes, particles are taken up in vesicular phagolysosomes. Intracellular phagosomal leakage, redox unbalance and ionic movements induced by toxic particles result in pro-IL-1β expression, inflammasome complex engagement, caspase-1 activation, pro-IL-1β cleavage, biologically-active IL-1β release and finally inflammatory cell death termed pyroptosis. In this review, we summarize the emerging signals and pathways involved in the expression, maturation and secretion of IL-1β during these responses to particles. We also highlight physicochemical characteristics of particles (size, surface and shape) which determine their capacity to induce inflammasome activation and IL-1β processing.

## Background

Interleukin-1β (IL-1β) is a highly active cytokine playing an essential role in inflammation and tissue damage. An abundant literature has depicted the details of IL-1β regulation and functions during infectious and inflammatory diseases. Various IL-1β-targeting agents such as IL-1 receptor antagonist, soluble decoy receptor and neutralizing monoclonal anti-IL-1β antibody have been used with success in several inflammatory diseases such as rheumatisms, diabetes and cancers in humans [1, 2]. The different components of the IL-1β signaling pathway have been largely elucidated and comprise the IL-1 type 1 receptor (IL-1RI), its co-receptor (IL-1RAcP) and the adaptor molecule (MyD88). Research on IL-1β has recently been revitalized by the discovery of the inflammasome machinery (constituted of the caspase-1 enzyme, ASC adaptor and NLRP sensor) which allows the release of mature IL-1β from the immature and inactive IL-1β pro-form. The transcription of the *IL1B* gene encoding pro-IL1β results from the activation of the transcription factors nuclear factor-kB (NFkB) and activator protein 1 (AP-1) [3, 4].

Intense research on the IL-1β/inflammasome axis has resulted in new concepts of cell death pathways and related signaling cascades. Activated caspase-1 cleaves gasdermin D to induce a lytic cell death, termed pyroptosis [5]. Pyroptosis represents a necrotic cell death that induces cell swelling and plasma membrane rupture with release of mature IL-1β and cytoplasmic content leading to an inflammatory reaction. IL-1β processing is, however, not specifically related to necrosis. Apoptosis-related caspase-8 also binds to inflammasome complexes, activates the NLRP3 inflammasome and processes IL-1β to induce apoptotic cell death. Similarly, RIPK3- and MLKL-dependent necroptotic signaling can activate the NLRP3 inflammasome to drive IL-1β inflammatory responses [5–7]. These findings strongly support the intimate interrelation of IL-1β processing and cell death. Finally, IL-1β release can occur independently of caspase1/inflammasome pathway through cleavage mechanisms related to cathepsins [8–10].

Interestingly, very early research on IL-1β revealed that monocyte-derived macrophages or tissue macrophages exposed to silica or asbestos particles strongly release IL-1β. Particle-induced inflammasome activation, IL-1β release and pyroptosis are still mostly described in macrophages and the exact cellular mechanisms that result in IL-1β processing after exposure to cytotoxic doses of particles have been mainly discovered in macrophages. However, other immune cells such as dendritic cells or monocytes are also able to release large amount of mature IL-1β. In addition, structural cells of various body compartments exposed to particles secrete, although to a lesser extent, mature IL-1β [11–18]. For instance, primary rat epithelial lung cells, cardiomyocytes, cardiofibroblasts and mesothelial cells also respond to particles by priming and activating inflammasome [13, 16, 19–21]. It remains however to explore whether the mechanisms of inflammasome activation and IL-1β maturation in these cells correspond to those defined in macrophages.

A major role of mature IL-1β in asbestosis and silicosis was proposed more than 30 years ago [22–24]. It is now well-established that active IL-1β serves as a primary initiating signal to coordinate the mobilization of immune cells to the damaged area caused by particles. Seminal studies in lung toxicology showed that IL-1β produced by particle-exposed macrophages induces, in concert with TNF-α, the production of chemokines by epithelial cells and mediates particle-induced neutrophil and macrophage influx and subsequent inflammatory lung responses [25–27]. The exact in vivo role of IL-1β in the development of chronic inflammation, fibrosis and cancer induced by particles has been reviewed in recent publications [28–31].

This review summarizes current knowledge on the main cellular signals responsible for the release of mature IL-1β after particle exposure. We first recapitulate the endogenous mediators (called signal 1) that prime the expression of the inactive pro-form of IL-1β (pro-IL-1β) by macrophages during the early response to particles. The second part delineates the intracellular events induced by particles (called signal 2) that result in NLRP3 inflammasome activation and IL-1β processing in macrophages. Finally, we highlight the physicochemical features of the particles which determine IL-1β processing.

### Priming cells to express pro-IL-1β: the role of alarmins and inflammatory cytokines

**Fig. 1:**
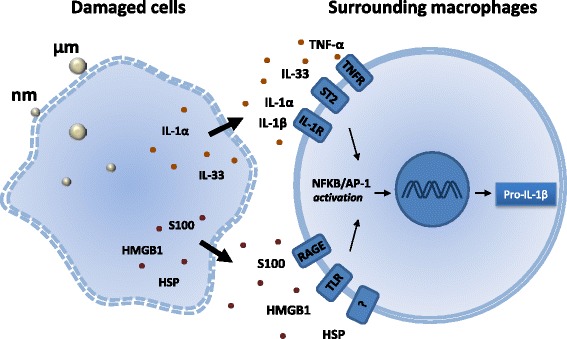
Processes involved in particle-induced pro-IL-1β expression. Pro-IL-1β expression requires intermediary mediators (signal 1). Silica-damaged macrophages or structural cells release intracellular proteins called alarmins that possess inflammatory activities once present in the extracellular environment. HGMB1 (High mobility group box-1), S100 and HSP (Heat shock proteins) proteins bind to multi-ligand receptors such as RAGE (Receptor for advanced glycation endproducts) or TLRs (Toll-like receptors) and stimulate the NFkB (transcription factors nuclear factor-kB)/AP-1 (Activator protein 1) pathway, leading to pro-IL-1β expression by surrounding macrophages. IL-1α and IL-33, two members of the IL-1 family, also pass across damaged cell membranes and bind their specific receptors, IL-1RI and ST2 (Interleukin 1 receptor-like 1), respectively. Additionally, other cytokines that are not classified as alarmins but known to promote pro-IL-1β production via NFkB/AP-1 activation (i.e., TNF-α and IL-1β itself) also participate in the expression of pro-IL-1β and synergize with alarmins

Activation of the IL-1 pathway requires first signals which comprise priming molecules inducing the transcription of pro-IL-1β through the NFkB/AP-1 signal transduction axis (signal 1). A variety of danger signals, also called alarmins, have been recognized as the first inflammatory signal elements strongly inducing pro-IL-1β expression. These molecules are usually sequestered inside homeostatic cells but released in the extracellular environment when the cell membrane is corrupted during necrosis, pyroptosis or if apoptotic bodies are not rapidly cleared and release their cytoplasmic content (secondary necrosis) (reviewed in [32]). The cytokines IL-1α, IL-33 and HMGB1 as well as certain heat shock (HSP) or S100 proteins are considered as potent alarmins during inflammation or immune responses to pathogens. They bind membrane receptors and trigger inflammatory pathways leading to NFkB or AP-1 activation and pro-IL-1β gene transcription. Besides alarmins, it is well known that IL-1β itself and TNF-α, another master pro-inflammatory cytokine, which are rapidly released by macrophages after exposure to particles, are considered as crucial priming factors (see Fig. 1).

1. **Interleukin-1**α

Expression of IL-1α is constitutive in diverse cells types in relation to the NFkB/AP-1 activation pathway (reviewed in [33, 34]). Akin IL-β, IL-1α is produced as a precursor. However, this pro-form is active and can bind IL-1RI to induce the production of inflammatory molecules. IL-1α lacks a secretory sequence signal and is released by an unconventional secretory pathway by simple diffusion across cell membrane upon membrane damage and necrosis or upon inflammasome activation. Several studies investigated IL-1α release in response to particles in LPS-primed cells [12, 35–37]. Less well described is the release of constitutive IL-1α cellular content. Primary rat lung epithelial cells exposed to ultrafine carbon black released IL-1α independently of a concomitant gene expression. The release of IL-1α preceded and amplified the production of other pro-inflammatory molecules such as IL-6 [16].

Fine (PM2.5) and, to a lesser extent, coarse particulates (PM10) from urban atmosphere induced IL-1α release from human bronchial epithelial cell line (BEAS-2B) [38]. We observed that IL-1α was released from cellular stocks present in primary macrophages or a macrophage cell line after exposure to silica or carbon nanotubes (CNT). Importantly, IL-1β release and neutrophil recruitment in the lung response to silica instillation were strongly reduced when mice received IL-1α neutralizing antibodies or in IL-1α-deficient mice [39, 40]. Similarly, IL-1β release in the peritoneal cavity following monosodium urate (MSU) injection was reduced in IL-1α-deficient mice [35]. These findings strongly support the view that IL-1α represents a major early signal released after particle exposure that allows the expression of IL-1β.

2. **HMGB1**

HMGB1 is constitutively expressed in all cells and can be released following cell necrosis or secreted by activated immune cells. Extracellular HMGB1, alone or complexed to other pro-inflammatory molecules can bind the RAGE receptor or TLRs, trigger the NFkB and AP-1 pathway and induce pro-inflammatory cytokine production [41, 42]. Particle-induced HMGB1 release has been documented in human macrophages and bronchial epithelial cell lines treated with silica or in asbestos-exposed mesothelial cells [14, 20, 21]. Passive and active release of HMGB1 has also been reported in cultures of an epithelial cell line or primary alveolar macrophages exposed to MWCNT [43]. The presence of HMGB1 in the extracellular environment increased IL-1β secretion by MWCNT-treated alveolar macrophages. Interestingly, inhibition of extracellular HMGB1 by neutralizing antibodies reduced MWCNT-induced IL-1β secretion and inflammation in vivo [43]. By using RAGE-deficient mice, Ramsgaard and colleagues also demonstrated that this receptor is involved in neutrophil influx following silica lung exposure [44]. Thus, HMGB1 is an additional important alarmin that mediates the expression of IL-1β.

3. **Interleukin-33**

Interleukin-33, a cytokine of the interleukin-1 family, is expressed by structural and inflammatory cells and, as a pro-form or after maturation, activates its receptor ST2 [45]. Similar to interleukin-1α and β, the precursor of this interleukin can be matured upon cleavage by several enzymes with different effects on its activity. Cleavage by caspase-1, 7 or 8 inactivates IL-33 whereas calpain and neutrophil- or mastocyte-derived proteases have the opposite effect by enhancing the alarmin activity [46]. IL-33 release in response to particle exposure has been reported in several studies. Alum has been shown to induce IL-33 release from THP-1 macrophages [47] and MWCNT induced IL-33 release in supernatant of an epithelial cell line and in broncho-alveolar lavage fluid of mice [48–51]. Although IL-33 is highly released after particle exposure, it is still unclear whether IL-33 is central to the induction of IL-1β expression after particle treatment.

4. **Other alarmins implicated in particle-induced priming**

S100A8/S100A9 proteins are constitutively expressed by phagocytes and released by non-classical pathways or by diffusion across cell membrane upon necrosis. Once in the extracellular environment, these mediators bind TLR4 or RAGE and activate the NFkB and AP-1 pathway (reviewed in [52, 53]). High levels of S100A8 and S100A9 were detected in BAL of rats exposed to diesel exhaust or ZnO particles [54, 55] and in lung tissue of mice exposed to SWCNT [56]. Heat shock protein (HSP) form a group of proteins that can bind various types of receptors, activate NFkB and trigger pro-inflammatory cytokine production ([57, 58] and reviewed in [59]). HSP60 release and subsequent TLR4 engagement have been implicated in the production of pro-IL-1β in monocytes exposed to polyethylene particles [60]. Despite their well-recognized role in sterile inflammation, S100 and HSP proteins have received little attention for their possible implication in IL-1β priming in the frame of particle-induced inflammation.

5. **Other cytokines implicated in particle-induced priming**

IL-1β regulates its own gene expression since its receptor IL-1RI is directly connected to the NFkB and AP-1 axis [61]. This suggests a possible autocrine loop in the production of pro-IL-1β during responses to particles. Interestingly, constitutive expression of pro-IL-1β has already been described in non-immune cells [14, 62–64]. The maturation of this constitutive pro-IL-1β may result from intracellular inflammasome mobilization or external proteases after pro-IL-1β diffusion [65–67]. Kono and colleagues observed that while IL-1β was crucial in silica-induced inflammatory response, caspase-1-deficient mice demonstrated only a limited reduction of inflammatory parameters in comparison to cathepsin C-deficient mice. Cathepsin C is necessary for activating serine proteases, and the authors postulated that its absence impaired extracellular IL-1β activation mediated by these proteases [8].

TNF-α is a powerful activator of NFkB/AP-1 and is known to induce IL-1β expression [68]. Under resting conditions, TNF-α translation is repressed in most cells [69] but rapidly restored under stress conditions [70]. In addition, a membrane-bound precursor of TNF-α can be processed by a TNF-α converting enzyme (TACE) to promptly generate secreted mature TNF-α [71]. Thus, TNF-α can be rapidly produced and released, independently of transcriptional induction, and can mediate early pro-IL-1β production. Release of TNF-α has been shown in response to different types of particles such as titanium nanoparticles, carbon nanotubes, polymethylmethacrylate particles, PM10 ambient particulate matter, wood smoke and traffic particles in vitro or in vivo [72–77]. Inhibition of TNF-α by a neutralizing antibody has been shown to reduce IL-1β production by A549 cells and human bronchial epithelial cells exposed to urban PM10 [75]. Interestingly, TNF-α promotes caspase-1 activation in response to silica [78].

Thus even if they are not considered to be constitutively expressed and stored such as classical alarmins, early release of IL-1β and TNF-α are involved in the priming step that allows immune cells to produce high amounts of IL-1β.

### Signals and mechanisms implicated in inflammasome processing of IL-1β in response to particles

**Fig. 2:**
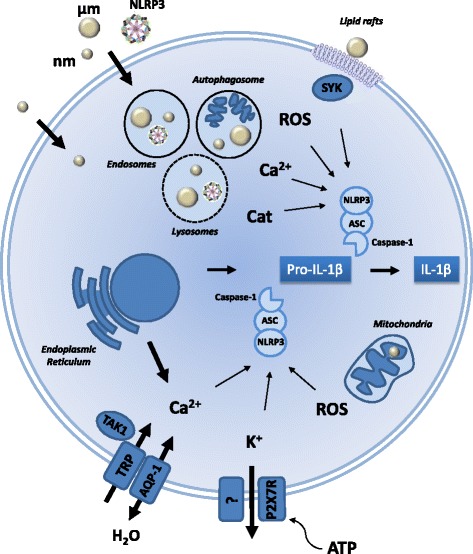
Cellular signals responsible for particle-induced inflammasome activation. Inflammasome activation after particle exposure results from various intracellular events (called signal 2) that are non-mutually exclusive. When endocytosed, nano- and micrometric-particles or exogenous NLRP3 complexes induce lysosomal destabilization and interfere with autophagy/mitophagy resulting in the release of ROS (Reactive oxygen species), cathepsins (Cat) or calcium in the cytosol. These vesicular leaking molecules promote the assembly of inflammasome components (NLRP3/ASC/Caspase-1) and subsequent IL-1β maturation from inactive pro-IL-1β form. Oxidative stress and active cathepsins modify undetermined protein structures which are recognized by the NLRP3 inflammasome. High calcium concentrations due to lysosomal but also endoplasmic reticulum release or extracellular influx via TRP (Transient receptor potential) calcium-channels affect mitochondria which release high amount of ROS. TAK1 (Tat-associated kinase), a kinase activated by increased intracellular calcium, is also implicated in inflammasome processing. Depletion in intracellular potassium is mandatory for inflammasome activation. Potassium cell efflux is indeed a necessary and sufficient signal for inflammasome activation and IL-1β processing. ATP release upon cell membrane damage permeates P2X7R (P2X purinoceptor 7) channels to potassium. Particle endocytosis is not systematically required and contact between cell membrane and particles resulting in the formation of lipid rafts is sufficient to trigger inflammasome engagement through SYK (Spleen tyrosine kinase) activation. The small size of nanoparticles allows them to cross biological membranes. Nanoparticles reach the cytosol even in absence of active endocytic process and may damage organelles such as mitochondria. Water movements through AQP (Aquaporin) 1 are necessary for inflammasome activation. Water channels are involved in inflammasome by regulating cytoskeleton rearrangement, ionic movements and TRP activation

The inflammasome is a multimeric complex mainly constituted by a sensor protein, the adapter protein Apoptosis-associated Speck-like protein containing CARD (ASC), and the cysteine protease caspase-1. Detection of a stimulus triggers a conformational change of sensor protein that leads to its oligomerization, recruitment of ASC through homotypic protein–protein interactions of the pyrin domains and subsequent recruitment and auto-activation of caspase-1, which in turn cleaves pro-IL-1β into its mature form [79]. NLPR3 is the sensor protein that has been mostly investigated in the context of particle-induced responses. NLRP3 is predominantly expressed in macrophages. NLRP3 inflammasome activation (also termed assembly or engagement) was first demonstrated by using MSU and calcium pyrophosphate dihydrate (CPPD) crystals [80]. Subsequently, a plethora of studies confirmed that diverse inflammatory particles are able to activate the NLRP3-inflammasome machinery. However, it appeared obvious that the NLRP3-protein complex cannot be directly triggered by particles themselves. Intensive investigations exploring indirect cellular events (signal 2) have led to propose at least four main processes accounting for inflammasome activation in response to particles: (1) lysosomal damage and subsequent lysosome content release, (2) modifications of intracellular ionic concentrations and localization, (3) intracellular redox unbalance and (4) organelle damage (summarized in Fig. 2).

1. **Critical role of lysosomal damage**

***Endocytosis***

The importance of endocytosis for particle-induced inflammasome engagement has been first demonstrated by using the actin polymerization inhibitors cytochalasin or lactrunculin in mononuclear and dendritic cells exposed to silica particles [81–84]. The essential role of actin-mediated endocytosis was further robustly established in response to various particles such as aluminum salt-constituted [36, 81, 85, 86], titanium dioxide (TiO2) [87] or polymeric particles [36, 88, 89] and asbestos or CNT fiber-shaped particles [90, 91]. However, cellular uptake of particles is not an obligatory step for the processing and the secretion of IL-1β after particle exposure. Indeed, a direct contact between particles and the cell membrane can be sufficient for activating the inflammasome complex in macrophages or dendritic cells. Macrophages significantly released IL-1β even if they were exposed to non-phagocytozed polymethylmethacrylate microspheres or MSU crystals [92, 93]. In addition, cell contact of non-phagocytable polystyrene beads [36] or surface-glued alum crystals also resulted in IL-1β secretion by dendritic cells without internalization [94]. In comparison with internalized particles, cell membrane-associated silica highly induced IL-1β release by macrophages [95]. Finally, lipid raft formation at cell membrane surface also leads to IL-1β secretion in response to large polymeric particles [92].

Thus, it appears that particle recognition and/or endocytosis are competent to cause inflammasome and IL-1β processing.

***Damage to lysosome***

Lysosomal rupture, induced by soluble destabilizing agents such as L-leucyl-L-leucine methyl ester (Leu-Leu-OMe), is sufficient for inflammasome activation [84]. A clear correlation has also been found between the lysosomolytic ability of particles and inflammasome activation potency. Silica particles accountable for a robust lysosomal destabilization induced IL-1β secretion [82, 96]. Implication of lysosomal leakage in inflammasome mobilization is now demonstrated in response to diverse silica particles in macrophages [82, 83, 95, 97] or dendritic cells [36]. Interestingly, the in vitro membranolytic activity of silica particles on red blood cells predicts the labilization of the phagolysosome, the activation of inflammasome and release of IL-1β [98].

Particles are endocytosed in vesicular phagosomes which then undergo fusion with lysosomes, forming phagolysosomes. The fusion of particle-containing vesicles with lysosomes leads to acidification and ROS production in an attempt to digest particles. Both biological processes can be implicated in lysosomal destabilization and inflammasome activation. Indeed, inhibition of endosomal acidification by bafilomycin A1 successfully reduced lysosomal leakage and the subsequent IL-1β production in macrophages or dendritic cells exposed to silica, titanium, alum or polymeric particles [36, 82–84, 87, 97]. Also, pretreatment of silica-exposed macrophages with the ROS scavenger butylated hydroxyanisole (BHA) strongly reduced endosomal rupture and subsequent IL-1β release [83].

Cytosolic leakage of cathepsin B after lysosomal damage also causes NLRP3 activation. The CA-074-Me cathepsin B inhibitor strongly affects IL-1β maturation induced by several fibrous and non-fibrous particles such as silica [35, 81, 83, 84, 97], titanium [12, 35, 87, 99], aluminum [36, 81, 85] or polymeric particles [36, 88], asbestos or CNT [37, 100]. However, the role of cathepsin B in particle-induced inflammasome activation is still a matter of debate. Indeed, cathepsin B-deficient bone marrow-derived macrophages (BMDMs) did not secrete less IL-1β and did not cleave caspase-1 less than WT cells in response to alum or silica [90, 93]. Similarly, cathepsin B deficiency did not reduce IL-1β release induced by liposomes in BMDM cultures whereas a cathepsin B inhibitor did [101]. This discrepancy could be explained by the lack of specificity of cathepsin inhibitors used in vitro or by the redundancy between cathepsins [102]. How cathepsin release could be implicated in caspase-1 and inflammasome activation is also not clear. Cathepsin B can directly process pro-caspase-1 [103] but a general intracellular protein degradation caused by cathepsin B release, that in turn induces caspase-1 activation, represents a more probable mechanism. In particular, cathepsin B leakage is also involved in mitochondrial damage [104] or potassium efflux [86], two processes that are mandatory in inflammasome activation (see below).

Finally, the lysosome contains high concentrations of ions and their release upon lysosomal membrane damage may also explain inflammasome activation. In particular, lysosomal damage and leakage can lead to deleterious cytosolic calcium concentrations and inflammasome activation [105].

In conclusion, particle-induced lysosomal damages induce multiple cellular perturbations that combine to maximize inflammasome activation in response to particles.

***Autophagy***

Lysosomal functions are not limited to digestion of endocytosed materials but also concern endogenous material elimination by a process called autophagy [106]. Autophagy is necessary to degrade and recycle damaged proteins and organelles, such as mitochondria. Inhibition of autophagy/mitophagy induces accumulation of ROS-generating damaged mitochondria and potentiates IL-1β processing in response to MSU crystals and soluble nigericin [107]. Particles impair normal autophagolysosome formation and induce inflammasome activation by the following events [106]. First, accumulation of non-destructible particles in phagolysosome leads to lysosome overload and prevents their fusion with autophagosomes [108]. Secondly, once in the cytosol, particles can bind to actin and impair cytoskeleton remodeling that is necessary for maturation and function of the autophagosome [109]. Finally, particles present in the cytosol can diffuse through the membrane and be incorporated in autophagic vesicles leading to their destabilization. For instance, nanoparticles can be incorporated to autophagic vesicles when ubiquitinated or associated with ubiquitinated proteins [110].

Altogether, these recent observations support the view that particles impair proper autophagic processes, favoring damaged mitochondria accumulation resulting in inflammasome processing.

2. **Ionic modifications**

***Potassium***

Reduced intracellular potassium concentration is mandatory for inflammasome activation. Reduction of intracellular potassium level induces a conformational change of NLRP3 allowing its activation [86, 111]. Additionally, potassium efflux could cause disruption of mitochondrial membrane potential [112] or ROS production [113]. Potassium efflux has been observed in response to silica exposure before IL-1β release and its inhibition reduced IL-1β and caspase-1 activation in response to silica, alum, silver or polymeric particles, asbestos or CNT in macrophages or dendritic cells [35, 36, 86, 89, 91, 101, 114–117]. How particle exposure leads to potassium efflux is still unknown. It has been suggested that plasma membrane damages or distortions caused by particle contact with cell surface may explain cellular potassium leakage. Activation of the P2X7R cation-channel in response to ATP binding has also been implicated in particle-induced potassium efflux and inflammasome activation. Riteau and colleagues demonstrated that following silica or alum phagocytosis and subsequent lysosomal leakage, cellular ATP is released in the extracellular environment where it can bind to P2X7R and activate the inflammasome [118]. IL-1β release in response to latex beads was also reduced in presence of apyrase (ATP diphosphohydrolase) or in P2X7R-deficient macrophages [89]. However, the implication of ATP and P2X7R in potassium efflux in the context of inhaled particles remains controversial since silica-induced IL-1β release by macrophages was not reduced by apyrase nor deficiency in P2X7R in other studies [117, 119, 120]. Thus, the exact mechanism by which potassium is released by particle-exposed cells still needs to be determined. Adenosine released by particle-exposed macrophages also activates the NLRP3 inflammasome by interacting with adenosine receptors and through cellular uptake by nucleoside transporters [121].

***Calcium***

While potassium efflux is a necessary and sufficient signal, modification of free cytosolic calcium concentrations has also been implicated in inflammasome activation in response to soluble activators [105, 122]. Few studies have investigated calcium modifications in cells exposed to particles and the role of this ion in inflammasome activation remains uncertain. It has been shown that alum crystals induce calcium mobilization from the endoplasmic reticulum that is required for NLRP3 inflammasome activation in BMDM cells [105]. Extracellular calcium influx also affects intracellular calcium balance. Exposure to silica and alum increased free cytosolic calcium concentration by an extracellular entry through ROS-activated TRPM2 channel (Transient receptor potential cation channel, subfamily M, member 2). Reduction of this influx by lowering extracellular calcium or suppressing TRPM2 channels leads to a partial decrease of IL-1β secretion [101, 105]. Calcium is implicated in multiple cellular functions and probably impacts the particle-induced inflammasome activation process at different levels. Indeed, actin polymerization and organelle trafficking necessary for phagolysosomal maturation are dependent of intracellular calcium movements. Thus, increased concentration of calcium could impact particle uptake and subsequent lysosomal damage. Potassium efflux necessary for inflammasome activation is also triggered by the activation of calcium-dependent potassium channels when cytosolic calcium concentrations are increased [123]. Finally, high levels of cellular calcium also induce mitochondrial dysfunction or trigger activation of TGF-β-activated kinase 1 (TAK1), both associated with inflammasome activation [105, 111].

In conclusion, it is probable that alteration of intracellular calcium homeostasis is involved in particle-induced inflammasome mobilization. However, the elucidation of the mechanism leading to this ionic dysregulation needs future investigations in cells exposed to particles.

3. **Oxidative stress**

Increased cellular production of ROS has been observed in response to most inflammasome activators. Interestingly, silica-induced ROS production was detected even in NLRP3-deficient macrophages, indicating that ROS production is upstream of inflammasome activation [114]. The use of ROS scavengers such as N-acetylcysteine or ebselen, a glutathione peroxidase mimic, efficiently reduced IL-1β release and caspase-1 activation in response to particles such as silica, alum or asbestos in dendritic or mesothelial cells [19, 35] and the deficiency in the ROS detoxifying protein thioredoxin (TRX) increased IL-1β maturation induced by silica and asbestos in macrophage cell lines [115]. TRX overexpression or treatment with recombinant TRX attenuated caspase-1 enzymatic activity and secretion of IL-1β in silica-exposed epithelial cell or macrophage cultures [124]. These data convincingly demonstrate that ROS production is a crucial event in inflammasome processing in response to particles.

In addition to ROS produced intrinsically by the particles themselves, the NADPH oxidase pathway and the damaged mitochondria also lead to intracellular ROS production. Upon particle phagocytosis, phagosome-associated NADPH oxidase produces ROS that could be released in the cytosol upon lysosomal leakage. Inhibition of NADPH oxidase by ROS inhibitors such as diphenyleneiodonium (DPI), ammonium pyrrolidinedithiocarbamate (APDC) or apocynin reduced IL-1β secretion or caspase-1 activation in response to silica, asbestos, CNT or titanium particles [37, 83, 87, 90, 101, 114, 115, 125]. The use of mice deficient in essential components of the membrane-associated phagocyte NADPH oxidase led, however, to confusing results. Cells lacking the p22phox expression had reduced inflammasome activation in response to asbestos whereas deficiency in gp91phox did not modify silica-induced inflammasome activation [84, 90, 115]. Interestingly, mitochondrial ROS production during inflammasome activation has also been demonstrated after silica and alum treatment in macrophages [85, 125]. Altogether, these studies indicate that the enzymatic and cellular pathways leading to ROS-induced inflammasome activation are diverse and might depend on particle physicochemical properties.

How ROS activate NLRP3 is still debated but it is postulated that proteins modified by oxidative stress directly bind NLRP3. The complex formed by the ROS detoxifying protein thioredoxin (TRX) and thioredoxin-interacting protein (TXNIP) has also been proposed to link ROS and NLRP3 activation. Under normal conditions, TXNIP is associated with TRX. However, the presence of free radicals oxidizes TRX that cannot bind TXNIP anymore. TXNIP then interacts with and activates NLRP3. TXNIP deficiency in antigen-presenting cells reduced caspase-1 activation and IL-1β release induced by silica, asbestos and alum [19, 107, 115]. The absence of TXNIP has also been shown to prevent IL-1β release in a model of MSU-induced peritonitis [107]. However, two studies failed to confirm requirement of TXNIP for inflammasome activation in response to silica and latex beads in BMDM [89, 126]. Finally, there is evidence that the sources of ROS are multiple and interconnected. Indeed, ROS released by particles or phagolysosomes directly or indirectly activate mitochondria to produce ROS. This amplification loop of free radical generation may explain why anti-oxidant cell defenses are supplanted after particle exposure and that the subsequent oxidative stress generated in cells activates inflammasome machinery.

4. **Organelle damage**

Mitochondrial damage has been proposed as an important event in NLRP3 inflammasome activation in response to soluble activators [107, 125] and has been associated with particle-induced inflammasome activation [89, 95, 116, 127]. Cathepsins, ROS and calcium release after lysosomal leakage participate to the mitochondrial damage induced by particles [104, 128]. Additionally, particles present in the cytosol after diffusion or lysosomal escape may directly affect normal mitochondrial function which may result in inflammasome activation [116]. Inhibition of damaged mitochondria clearance in BMDM exposed to latex beads leads to increased IL-1β release, probably due to uncontrolled ROS release [89]. Under resting conditions NLRP3 localizes to endoplasmic reticulum (ER) structures in THP-1 macrophages but upon exposure to inflammasome-activating crystals such as alum, NLRP3/ER complexes and ASC are relocalized to mitochondria. Authors proposed that mitochondria recruit inflammasome components and favor their interactions. Additionally, voltage-dependent anion-selective channel protein 1 (VDAC1), a channel present at the mitochondrial membrane and controlling calcium transfer from ER, was implicated in caspase-1 activation and IL-1β release in response to silica and alum, possibly via ROS production [107]. Finally, cardiolipin, a mitochondrial-specific phospholipid, translocates from the inner to the outer mitochondrial membrane and binds NLRP3, explaining why inflammasome co-localizes with mitochondria. This interaction then leads to caspase-1-mediated IL-1β cleavage [125].

5. **New mechanisms of particles-induced inflammasome activation**

Macrophage swelling and subsequent regulatory volume decrease have been associated with NLRP3 inflammasome activation and IL-1β maturation in response to different stimuli [35, 111, 129]. Interestingly, cell volume modifications have been reported in the past in response to particle endocytosis [37, 130, 131]. Recently, we demonstrated that water movements through aquaporin (AQP), in particular AQP1, are necessary for inflammasome activation in response to particles in murine macrophages. AQP is implicated in swelling and shrinkage of the cell to restore its homeostatic volume [132]. Several mechanisms could explain the role of AQP in inflammasome mobilization. AQP mediates cytoskeleton rearrangement [133] necessary for particle endocytosis, intracellular vesicular trafficking and inflammasome components localization with filamentous actin [134–136]. The reduction of AQP-controlled water flux and volume alterations probably affect potassium and calcium movements which are necessary for particle-induced inflammasome activation. AQP could be necessary for calcium-channel TRP activation [137, 138].

The ubiquitination process allows addressing protein to the proteasome for their elimination, and regulates inflammasome activity by targeting the degradation of inflammasome components by autophagy [139]. For instance, NLRP3 ubiquitination reduced inflammasome activation in response to various activators such as silica crystals [140]. On the other hand, it has been shown that the linear ubiquitination of ASC is necessary for silica-induced inflammasome activation in BMDM cells [141]. Ubiquitination may thus repress or promote the particle-induced inflammasome machinery according to the ubiquitinated protein and ubiquitination process considered.

Various kinases have been implicated in the pathway leading to IL-1β secretion after particle exposure [16, 35, 142, 143]. In particular, Spleen tyrosine kinase (SYK), a kinase regulating endocytosis and actin remodeling processes, has been involved in inflammasome activation in response to polymeric particles, silica, alum, asbestos and carbon nanotubes [37, 81, 92, 94]. In dendritic cells, contact between cell membrane and uric crystals results in membrane lipid alteration that induces activation of SYK and inflammasome activation [92, 94]. TAK1, a kinase involved in TLR signaling and activated by intracellular Ca2+ variations, has also been involved in inflammasome processing in response to ATP and osmotic stress [111, 144]. Interestingly, this kinase has also been involved in inflammasome processing consecutive to lysosomal rupture induced by Leu-Leu-OMe or uric crystals [145]. The GTPase effector Rho-kinases (ROCK1, and 2) regulating cytoskeleton and phagocytosis have also been involved in fibrous particle-induced inflammasome responses in THP-1 cells [146].

Recently, different groups demonstrated that inflammasome activation leads to the release of ASC and NLRP3 that form functional oligomeric inflammasome particles. These complexes can be subsequently phagocytized by surrounding macrophages and trigger lysosomal damage and inflammasome activation. Additionally, ASC-NLRP3 complexes also form functional inflammasomes in bystander macrophages after being internalized [147–149].

### Physicochemical characteristics of particles determining inflammasome activation

Contrary to water soluble agents, the toxicity of particles cannot solely be determined by chemical composition and molecular structure. Various characteristics such as (1) size, (2) surface curvature and (3) surface area or shape strongly affect particle internalization, intracellular localization, cell responses and IL-1β processing. A summary of studies considering the influence of particle characteristics on inflammasome activation and IL-1β release is provided in Tables 1, 2 and 3.

**Table 1:** Effects of particle size on the ability of cultured phagocytes to process and release IL-1β

Size						
Chemical composition (doses)	Primary size (nm)	Hydrodynamic diameter (nm)	IL-1β release	Identified mechanisms	Cell type	Reference
Amorphous silica (100–1000 μg/ml)	20	295	++++	Lysosomal acidification and cathepsin B activity	Macrophages	[97]
	67	91	+++	Lysosomal acidification and cathepsin B activity		
	369	531	++	N.a.		
	500 - 10000	342 - 5560	+	Lysosomal acidification and cathepsin B activity		
	30	/	++++	Actin-mediated endocytosis and lysosomal acidification	Macrophages	[82]
	100	/	+++	Actin-mediated endocytosis and lysosomal acidification		
	300	/	+++	Actin-mediated endocytosis and lysosomal acidification		
	1000	/	+++	Actin-mediated endocytosis and lysosomal acidification		
	3000	/	++	Actin-mediated endocytosis and lysosomal acidification		
	10000	/	+/−	Lysosomal acidification		
Carbon black (62–687 μg/ml)	53,7	235	++	N.a.	Monocytes	[165]
	525	636	+	N.a.		
	14	/	++	N.a.	Monocytes	[166]
	260	/	-	N.a.		
Silver (0.15 – 0.9 μg/ml)	5	/	++	Potassium efflux and oxydative stress	Monocytes	[116]
	28	/	+	Potassium efflux and oxydative stress		
	100	/	-	N.a.		
Polystyrene (120 – 3000 μg/ml)	1000	/	++++	N.a.	Dendritic cells	[36]
	430	/	+++	Actin-mediated endocytosis, lysosomal acidification cathepsin B activity and potassium efflux		
	10 000	/	++	N.a.		
	32 000	/	+	N.a.		

The smallest and fiber- or needle-like particles are particularly active to induce IL-1β release. Surface area properties and reactivity also govern inflammasome/IL-1β activation. Physical or chemical treatments aiming to reduce surface reactivity can control inflammogenicity of particles

*N.a.* not assessed, *N.r.* not relevant

**Table 2:** Effects of particle surface on the ability of cultured phagocytes to process and release IL-1β

Surface						
Chemical composition (doses)	Surface characteristics	Radicals produced at cell surface	IL-1β release	Identified mechanisms	Cell type	Reference
Amorphous silica (50–200 μg/ml)	Midly -	+	++	Actin-mediated endocytosis, lysosomal acidification and cathepsin B activity, oxidative stress	Macrophages	[83]
	- (−COOH)	/	+	Actin-mediated endocytosis, lysosomal acidification and cathepsin B activity, oxidative stress		
	+ (−NH_2_)	/	-	N. r.		
	Silanol +++	+++	+++	N.a.	Macrophages	[95]
	Silanol +++	++	++	Independent of entry and cathepsin B release		
	Silanol ++	+	+	N.a.		
	Silanol +	+	-	N.r.		
Polystyrene (100 μg/ml)	+ (−NH_2_)	/	+	Oxidative stress	Macrophages	[127]
	- (−COOH)	/	-	N.r.		
	/	/	-	N.r.		
Aluminum oxyhydroxyde (500 μg/ml)	OH- +++++	++++	++++	Actin-mediated endocytosis, lysosomal acidification and cathepsin B activity, oxidative stress	Monocytes and macrophages	[85]
	OH- ++++	+++	+++	Actin-mediated endocytosis and cathepsin B activity, oxidative stress		
	OH- ++++	++	++	Actin-mediated endocytosis and cathepsin B activity, oxidative stress		
	OH- ++	+	+	Actin-mediated endocytosis and cathepsin B activity, oxidative stress		
	OH- +	+	+/−	Oxidative stress (actin-mediated endocytosis and cathepsin B activity not convincing)		
MWCNT (10–100 μg/ml)	Raw	/	+++	Lysosomal damage and cathepsin B activity	Macrophages	[100]
	Purified (less Ni contamination)	/	++	Lysosomal damage and cathepsin B activity		
	- (−COOH)	/	+	Cathepsin B activity		

The smallest and fiber- or needle-like particles are particularly active to induce IL-1β release. Surface area properties and reactivity also govern inflammasome/IL-1β activation. Physical or chemical treatments aiming to reduce surface reactivity can control inflammogenicity of particles

*N.a.* not assessed, *N.r.* not relevant

**Table 3:** Effects of particle shape on the ability of cultured phagocytes to process and release IL-1β

Shape						
Chemical composition (doses)	Shape	Length/width (nm) (ratio)	IL-1β release	Identified mechanisms	Cell type	Reference
Titanium rutile (20–500 μg/ml)	Spicula	40/10 (4)	++	Actin-mediated endocytosis, lipid raft, lysosomal acidification and cathepsin B activity, oxidative stress	Macrophages	[87]
	Spheric	30-40 (≈1)	+	Actin-mediated endocytosis, lipid raft, lysosomal acidification and cathepsin B activity, oxidative stress		
Poly(ethylene oxide) (3–100 μg/ml)	Spherical with budding	N.r.	++	Actin-mediated endocytosis and cathepsin B activity	Macrophages	[88]
	Spherical	N.r.	+	Actin-mediated endocytosis and Cathepsin B activity		
	Spherical	N.r.	-	N.r.		
Gold (2–10 μg/ml)	Rod	40/10 (4)	+	N.a.	Macrophages	[164]
	Spherical	40 (≈1)	-	N.a.		
	Cubic	40/40/40 (≈1)	-	N.a.		
Carbon (2–100 μg/ml)	Needle-like MWCNT	13 000/>50(<260)	+	Cathepsin B activity, oxydative stress, src/syk pathway and P2X7R activity	Macrophages	[37]
	Tangled MWCNT	10 000–50 000/8 – 15(667–6250)	-	N.r.		
	Short MWCNT	1000 - 10 000/0,5 - 20/(50–20000)	-	N.r.		
	Needle-like MWCNT	4150/150 (28)	+++	Actin-mediated endocytosis	Macrophages	[163]
	Needle-like HTCFNW	7600/586 (13)	++	N.a.		
	Needle-like HTCFNW	1800/365 (5)	+	Actin-mediated endocytosis		

The smallest and fiber- or needle-like particles are particularly active to induce IL-1β release. Surface area properties and reactivity also govern inflammasome/IL-1β activation. Physical or chemical treatments aiming to reduce surface reactivity can control inflammogenicity of particles

*N.a.* not assessed, *N.r.* not relevant

1. **Size**

Particle size is decisive for the processing and release of biologically-active IL-1β by phagocytic cells. This notion results from recent studies showing that nanoparticles possess a strong capacity to induce IL-1β release. BMDM exposed to amorphous silica nanoparticles with size ranging from 30 nm to 10 μm released more IL-1β in response to the smallest particles (30–1000 nm > 3 μm > 10 μm, when compared on a mass-based dose). Lysosomal damage and not internalization or actin polymerization explained these size-related differences [82]. Another study confirmed that, when compared on a mass-based dose, nanometric amorphous silica particles induced more IL-1β release by macrophages than their submicrometric counterparts (50 nm vs 500 nm) [97]. BMDM and primary glial cells exposed to similar mass doses of latex beads released more IL-1β in response to 20 nm than 1 μm size particles. In this study, inflammasome activation was attributed to lysosomal destabilization and cathepsin B release for 20 nm particles and to ROS production and mitochondrial damage for 1 μm particles. Additionally, inflammasome activation by the 20 nm particles was associated with their capacity to induce cellular damage and ATP release [89]. In dendritic cells, IL-1β release after polystyrene particle exposure (mass dose) was higher in response to 430 nm and 1 μm than to the 10 or 32 μm particles. In this model, small polystyrene particles were more efficiently internalized in comparison with larger particles [36]. Silver nanoparticles of 5, 28 and 100 nm were all internalized in monocytes but only 5 and 28 nm induced vesicular damage with ROS production and IL-1β release [116]. The relatively low capacity of micrometric particles to activate the inflammasome appears related with a lower endocytosis and lysosomal damage. It is also important to emphasize that the small size of nanoparticles allows them to reach intracellular compartments such as mitochondria [150] or to bind proteins such as actin [109]. Simple diffusion of nanomaterials across the cell membrane can be sufficient to activate inflammasome in keratinocytes [12].

Altogether, these data suggest that smallest particles are more potent, on a mass-based dose, to activate inflammasome in phagocytic cells.

There are, however, some reported exceptions: THP-1 cells failed to release IL-1β in response to amorphous silica particles below 1 μm [83] and spherical polystyrene particles of 7–8 μm induced inflammasome processing in macrophages whereas 0.5 and 1 μm did not [88]. Long fiber-shaped carbon, TiO_2_ or CeO_2_ nanoparticles induced more IL-1β release than shorter ones in macrophages [37, 151–153]. Importantly, the impact of particle size differs according to the cell type considered and their endocytic capacities. In non-phagocytic cells such as keratinocytes, only nanoparticles but not micrometric particles are internalized and induce inflammasome activity [12, 107].

Additionally, aggregation or agglomeration state of particles (mainly nanoparticles) modifies particle diameter, curvature, density, endocytosis and thus subsequent inflammasome activation. Surface area available for particle reactivity can also be modified upon aggregation/agglomeration (see below). Unfortunately, no study formally and directly assessed the impact of aggregation on IL-1β release. However, aluminum nanoplates and polyhedron of different aggregate sizes (hydrodynamic diameter of 93 nm and 333 nm, respectively) but with similar primary size, specific surface area and surface reactivity induced similar level of inflammasome processing suggesting minor impact of aggregation [85]. Our group also demonstrated that aggregation in the nanorange did not modify the cytotoxic effect of silica nanoparticles in macrophages [154].

2. **Surface area**

On a mass basis, nanoparticles were frequently claimed to be more cytotoxic, but, once normalized by particle number or surface area, this difference was blunted [155, 156]. This issue has also been addressed in the frame of inflammasome activation. Expressed in mass concentration, 50 nm amorphous silica particles induced more IL-1β release than 500 nm in macrophages but when dose was expressed in total surface area this trend was reversed [97]. Similarly, differences in IL-1β levels released by BMDM in response to amorphous silica particles ranging from 30 to 1000 nm were dampened upon normalization by particle number and almost abolished once expressed in total surface area. However, in this study, particles over 1 μm of diameter were less internalized and macrophages released lower amounts of IL-1β after surface area normalization [82]. Altogether, these data suggest that surface area highly determines inflammasome activity of internalized particles.

Reactive groups present at particle surface can also affect the ability of particles to induce inflammasome activation. It is already known that silanol groups present on the surface of silica particles are associated with their toxic activity [157, 158]. Interestingly, reduction of silanol surface density by heating was associated with a decreased IL-1β secretion by THP-1 cells. Subsequent rehydration restored initial silanol concentration on particle surface together with IL-1β processing and release, demonstrating their implication in inflammasome activation. However, in the same study, particles with similar size, surface area and silanol characteristics but with different synthesis process presented different levels of inflammasome activation capacity, probably in relation with their ability to produce free radicals under acellular conditions [95]. The hydroxyl surface amount also affected IL-1β release in response to aluminum nanoparticles in THP-1 cultures. Indeed, particle presenting higher amounts of surface hydroxyl induced more inflammasome processing. This effect was attributed by the authors to an increased capacity of hydroxyl-rich particles to induce ROS production and lysosomal damage [85]. Surface metal contaminants can also exacerbate IL-1β cleavage by catalyzing surface radical production on particles. Chelation of iron reduced IL-1β maturation after asbestos exposure probably by limiting oxidative stress [115]. Oxidative dissolution of silver nanoparticles upon lysosomal acidification leads to Ag ion release, ROS byproducts, mitochondrial-derived ROS and ultimately inflammasome activation in THP-1 cells [159, 160]. Nickel contamination was also implicated in CNT-induced inflammasome activtion in mouse primary alveolar macrophages [161].

Importantly, functionalization of amorphous silica particles with various surface groups (−COOH, −NH2, −SO3H and -CHO) reduced particle-induced IL-1β release by THP-1 cells. These functionalizations reduced endosomal rupture, cathepsin B leakage and ROS production by limiting the access of surface silanol groups without affecting particle uptake [83]. Carboxylic functionalization of MWCNT completely inhibited IL-1β release by macrophages after clearing nickel contaminant [100, 151]. Carboxylation of titanium nanobelts also reduced IL-1β release by mouse alveolar macrophages and THP-1 cells, probably by reducing lysosomal damage [162].

Particle surface charge appears as another parameter important for inflammasome processing. Polystyrene beads induced lysosomal damage, cathepsin B release, mitochondrial ROS production and IL-1β release solely after amino-functionalization. Amino-functionalization induced lysosomal destabilization consistent with the proton sponge theory. The amine present at particle surface traps protons. Consequently, proton pump activity is increased and each proton that enters the lysosome is accompanied by one chloride ion and one water molecule. This influx of ions and water leads to lysosomal swelling and destabilization as well as IL-1β release [127].

In conclusion, the surface reactivity determines the ability of particles to induce lysosomal membrane destabilization and inflammasome activation. This effect results from the surface characteristics, chemical composition or contamination. Consequently, treatments altering particle surface reactivity by eliminating reactive groups or contaminants can be useful in order to reduce particle inflammogenicity.

3. **Shape**

By affecting internalization and lysosomal stability, the shape of particles is another important parameter which determines the activity of particles on the inflammasome machinery. In particular the high length/width ratio appears important in inflammasome activation by fibers. Inert in THP-1 cells, CeO2 nanocubes or nanorods activate the inflammasome when their length is increased. Indeed, these high length/width aspect ratio particles were able to destabilize lysosomal membrane leading to cathepsin B release and subsequent inflammasome activation [153]. Long TiO2 nanobelts induced more inflammasome activation than short nanobelts and nanospheres in alveolar macrophages. This activity was also linked to lysosomal destabilization and cathepsin B release [152]. Similarly, spiculated TiO2 particles induced stronger IL-1β release by macrophages than spherical nanoparticles with similar size [87]. Long well-dispersed carbon nanotubes as well as needle-like calcined fullerene nanowhiskers (HTCFNW) activate more intensively inflammasome than their shorter counterpart [163]. Similarly, needle-like carbon nanotubes are more active than spherical carbon black nanoparticles and shorter nanotubes [37]. Among spherical and rod-shaped gold nanoparticles in the same size range (20 and 40 nm diameter sphere and 10 nm witdh/40 nm length rods), only rods were able to induce IL-1β release, even if all were endocytosed and both 20 nm spheres and rods escaped lysosomes [164]. Curvature is also an important particle characteristic for inflammasome activation. Spherical polymeric particles composed of budding with combination of high positive and negative surface curvature released more IL-1β than smooth particles of the same size (7–8 μm). This effect was correlated with the level of internalized or associated budding particles [88].

Altogether, these data indicate that the shape of particles is also a major parameter determining particle-induced inflammasome activation. Particles with an aspect ratio close to one are particularly less efficient to induce inflammasome activation than the longer ones.

## Conclusions

After particle exposure, alarmins retained intracellularly as preexisting stocks in lung resident cells and additional early pro-inflammatory cytokines are released into the extracellular milieu. These first inflammatory mediators (signal 1, Fig. 1) are potent activating stimuli required for macrophages, meso- and epithelial cells to express the biologically inactive precursor IL-1β (pro-IL-1β). This form is subsequently cleaved by particle-induced inflammasome activation prior its secretion as mature and bioactive IL-1β.

The processes leading to inflammasome activation and IL-1β maturation are initiated following lysosomal destabilization and leakage of enzymes, ions or ROS (signal 2, Fig. 2). These cellular events can result in mitochondrial damage, crucial to NLRP3 and inflammasome activation. Physicochemical characteristics of particles such as size and shape are decisive for particle internalization and lysosomal alteration. The smallest and fiber- or needle-like particles are particularly active to induce inflammasome activation. Surface area properties and reactivity also govern lysosomal damage and subsequent inflammasome/IL-1β processing. Physical or chemical treatments aiming to reduce surface reactivity can control inflammogenicity of particles. Nanoparticles can reach intracellular compartments and trigger metabolic processes, and induce toxicity and inflammasome activation by new pathways that are still to delineate. The observation that diverse particles are able to activate the inflammasome machinery allows considering the IL-1-related machinery as a new and crucial pathogenic pathway in particle toxicology.

## Abbreviations

AM, alveolar macrophages; AP-1, activator protein 1; AQP, aquaporin 1; ASC, apoptosis-associated speck-like protein containing CARD; ATP, adenosine triphosphate; BMDM, bone marrow-derived macrophages; Casp-1, cysteine protease caspase-1; Cat, cathepsin; CB, carbon black; CeO2, cerium oxide; CNT, carbon nanotube; CPPD, calcium pyrophosphate dihydrate; HGMB1, high mobility group box-1; HSP, heat shock protein; IL, interleukin; LPS, lipopolysaccharides; MLKL, mixed lineage kinase domain-like; MSU, monosodium urate crystals; MW, multi walled; Myd88, myeloid differentiation primary response gene 88; NF-kB, nuclear factor-kappa B; NLR, NOD-like receptor or nucleotide-binding oligomerization domain receptor; NLRP3, NLR family pyrin domain contain 3; P2X7R, P2X purinoceptor 7; PM, particulate matter; RAGE, receptor for advanced glycation end products; RIPK3, receptor interacting serine/threonine kinase 3; ROS, reactive oxygen species; ST2, interleukin 1 receptor-like 1; SW, single walled; SYK, Spleen tyrosine kinase; TACE, TNF-α converting enzyme; TAK1, Tat-associated kinase or TGF-β-activated kinase 1; THP-1, human monocytic cell line; TiO2, titanium dioxide; TLRs, toll-like receptors; TNF, tumor necrosis factor; TRP, transient receptor potential; TRPM2, transient receptor potential cation channel, subfamily M, member 2; TRX, detoxifying protein thioredoxin; TXNIP, thioredoxin-interacting protein
